# Acute Malignant Ascites Revealing Mucinous Adenocarcinoma of the Gallbladder: A Rare Case in a Recent Migrant

**DOI:** 10.7759/cureus.107409

**Published:** 2026-04-20

**Authors:** Juan R Santos-Rivera, Juan G Jimenez Garcia, Guillermo Izquierdo-Pretel

**Affiliations:** 1 Internal Medicine, Ponce Health Sciences University, Ponce, PRI; 2 Internal Medicine, Florida International University, Herbert Wertheim College of Medicine, Miami, USA; 3 Hospital Medicine, Jackson Memorial Hospital, Miami, USA

**Keywords:** advanced gallbladder cancer, gallbladder cancer, gallbladder histopathology, malignant ascites, metastatic gallbladder mucinous adenocarcinoma

## Abstract

Mucinous adenocarcinoma of the gallbladder is a rare and aggressive histologic subtype, most often diagnosed at an advanced stage due to its nonspecific clinical presentation. We report the case of a 50-year-old male who recently migrated from Venezuela and presented with progressive abdominal distension, peripheral edema, and systemic symptoms. Diagnostic paracentesis revealed a low serum-ascites albumin gradient (SAAG), raising concern for malignancy. Cross-sectional imaging demonstrated a large gallbladder mass with hepatic metastases, and histopathologic analysis from liver biopsy confirmed stage IV mucinous adenocarcinoma. Despite initially preserved functional status, the patient experienced rapid clinical deterioration, developing duodenal obstruction, sepsis, and multi-organ failure, leading to death within 48 hours of readmission.

This case highlights the aggressive clinical course and diagnostic challenges of mucinous gallbladder carcinoma. It underscores the importance of early tissue diagnosis in patients with low-SAAG ascites, even when initial cytology is inconclusive, and reinforces the need for heightened clinical suspicion for underlying malignancy in patients presenting with unexplained ascites.

## Introduction

Gallbladder carcinoma, although uncommon, accounts for approximately 50% of all biliary tract malignancies [1]. Mucinous adenocarcinoma represents a rare histopathologic subtype. Chronic inflammation is recognized as the principal pathogenic driver in gallbladder adenocarcinoma, and many clinical manifestations arise from this underlying process [2]. Early-stage disease is frequently asymptomatic, whereas advanced disease typically presents with nonspecific symptoms such as abdominal pain, nausea, anorexia, weight loss, weakness, and jaundice, contributing to delayed diagnosis.

Persistent inflammation is strongly associated with several established risk factors for malignant transformation. Cholelithiasis (gallstones) demonstrates the strongest correlation with gallbladder carcinoma, while gallbladder calcification and polyps further increase risk. Residence in regions endemic for *Salmonella Typhi *and *Helicobacter pylori* has also been linked to increased incidence [3]. These infections are prevalent in Venezuela, the country of origin of the patient described in this report [4,5]. Globally, gallbladder cancer incidence is highest in South America and South Asia, with Venezuela considered a moderate-to-high incidence region. Together, these epidemiologic patterns emphasize the importance of heightened clinical vigilance and tailored screening strategies in immigrant and underserved populations exposed to region-specific carcinogenic factors [6].

Mucinous adenocarcinoma of the gallbladder is defined histologically by the presence of more than 50% extracellular mucin within the tumor. It accounts for approximately 2.5% of gallbladder carcinomas and is associated with poorer outcomes than other subtypes, largely because it is often identified at a metastatic stage [7]. Gallbladder cancer is staged from 0 (localized) to IV (metastatic) [8], and the liver, regional lymph nodes, and peritoneal surfaces represent the most common sites of spread [9]. The absence of distinctive clinical features further complicates early recognition. When diagnosed at a resectable stage, cholecystectomy remains the cornerstone of treatment.

We present this case to expand the limited literature on mucinous adenocarcinoma of the gallbladder and to highlight the diagnostic and therapeutic challenges inherent to this subtype. This report illustrates rapid clinical progression, extensive metastatic disease at presentation, and limited treatment responsiveness, particularly in the context of a recently arrived migrant with potential exposure to endemic risk factors. By underscoring these features, we aim to reinforce the need for earlier recognition and focused evaluation of patients from high-risk regions.

## Case presentation

Initial presentation

In February 2024, a 50-year-old male with no significant past medical history presented to a tertiary care hospital with a four-week history of progressive abdominal distension, bilateral lower extremity edema, and low back pain. He had recently migrated from Venezuela, leaving in September 2023, traveling through Central America, and arriving in the United States in November 2023.

He denied prior known liver disease, alcohol misuse, or malignancy. At presentation, he reported progressive weakness but remained independent in activities of daily living.

On physical examination, vital signs were stable. Abdominal examination revealed marked distension with a positive fluid wave consistent with large-volume ascites. Mild scleral icterus was noted. There was bilateral pitting edema of the lower extremities. No focal abdominal tenderness was appreciated.

Diagnostic workup

Initial laboratory evaluation (Table 1) demonstrated a markedly elevated carcinoembryonic antigen (CEA) level, while liver function tests, CA 19-9, ceruloplasmin, and autoimmune panel were within normal limits. Diagnostic paracentesis yielded four liters of ascitic fluid. The serum-ascites albumin gradient (SAAG) was low, supporting a non-portal hypertensive etiology consistent with malignant ascites. Cytologic analysis revealed poorly differentiated carcinoma.

**Table 1:** Summary of pertinent laboratory findings at initial evaluation

Parameter	Value	Reference range	Interpretation
Carcinoembryonic antigen (CEA)	Markedly increased	≤ 5.0 ng/mL	Elevated
Liver function tests	Within normal limits	—	Normal
Ceruloplasmin	Within normal limits	—	Normal
CA 19-9	Within normal limits	≤ 37 U/mL	Normal
Autoimmune panel	Within normal limits	—	Negative
SAAG (serum-ascites albumin gradient)	Negative gradient	≥ 1.1 g/dL (portal HTN)	Suggestive of malignancy

**Figure 1:**
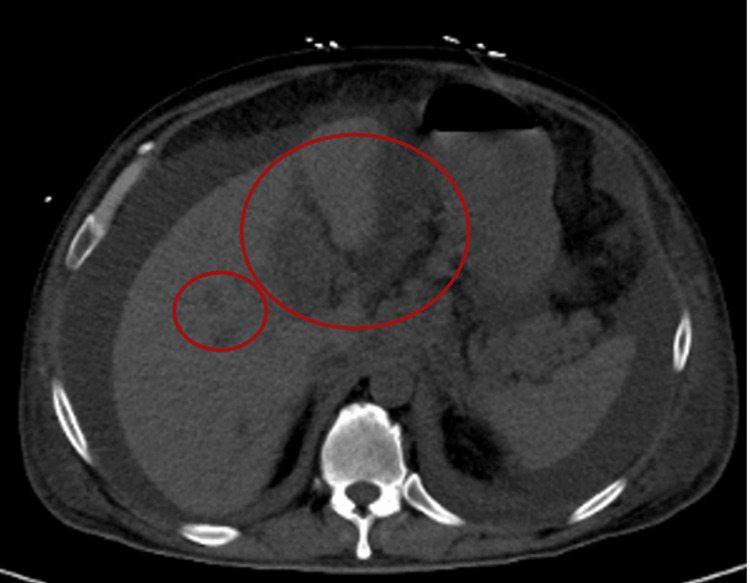
CT image showing a complex mass involving the liver and gallbladder (larger red circle) as well as additional hepatic lesions (smaller red circle), concerning for metastatic disease.

**Figure 2:**
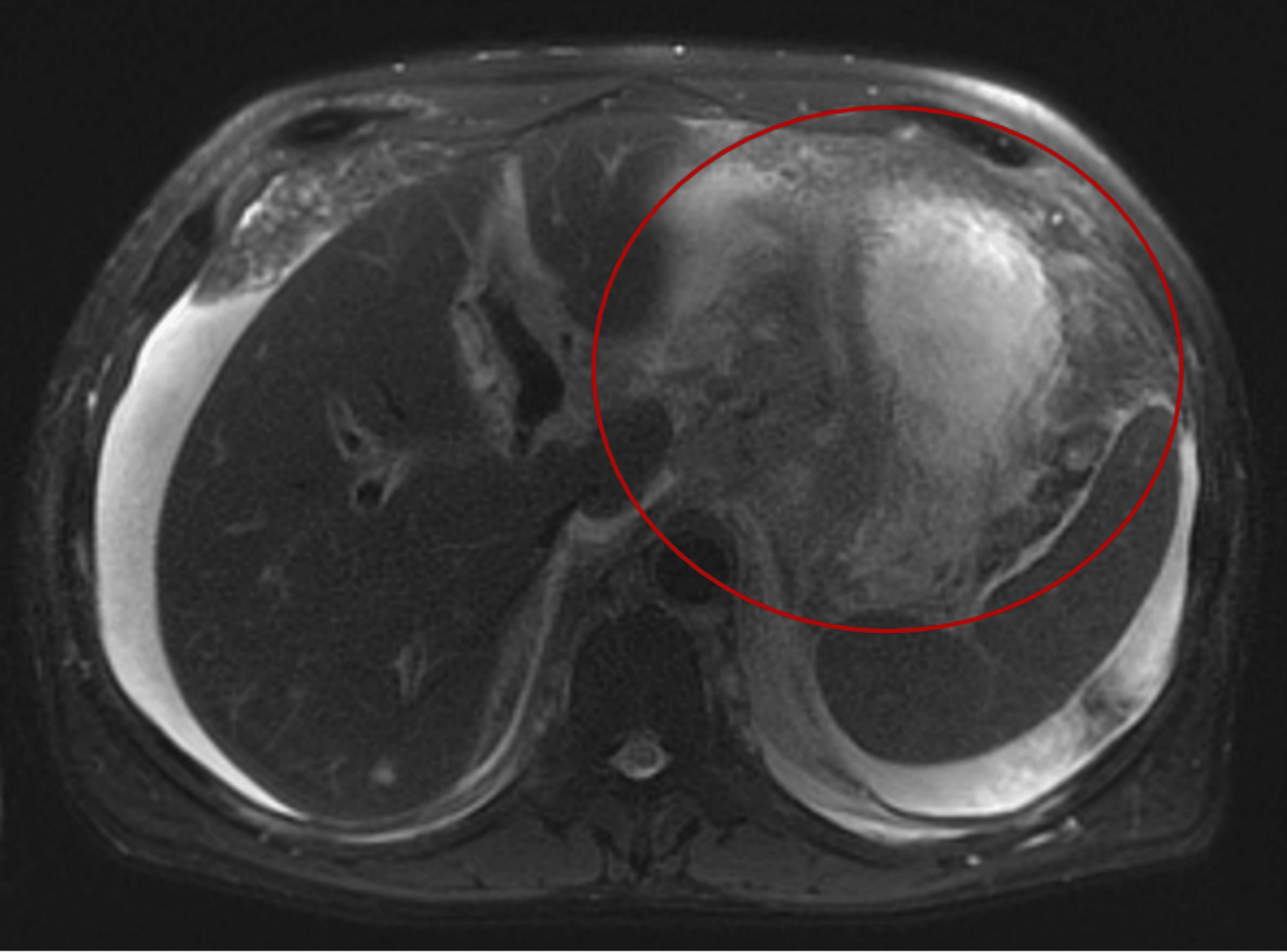
MRI of the abdomen demonstrating a large, heterogeneously enhancing mass involving the gallbladder and adjacent liver parenchyma (red circle). Differential considerations include gallbladder carcinoma and cholangiocarcinoma.

Computed tomography (CT) of the abdomen and pelvis without contrast demonstrated a complex cystic mass centered in the gallbladder with multiple hypodense hepatic lesions and mesenteric and retroperitoneal lymphadenopathy (Figure 1). Magnetic resonance imaging (MRI) of the abdomen with and without contrast further characterized a 10.3 × 7.5 × 8.8 cm heterogeneously enhancing mass involving the gallbladder with direct invasion into adjacent hepatic parenchyma (Figure 2). The imaging findings were concerning for advanced primary gallbladder carcinoma with hepatic metastases.

Given the presence of malignant ascites and hepatic lesions, differential considerations at this stage included primary hepatobiliary malignancy, metastatic gastrointestinal carcinoma, and cholangiocarcinoma.

**Figure 3:**
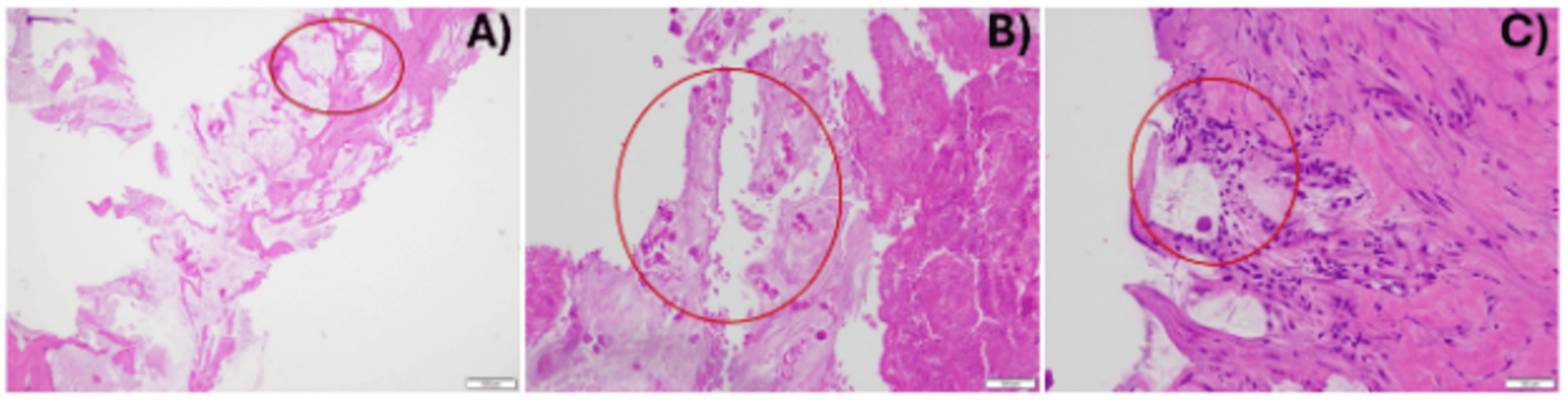
Histopathology from the liver biopsy specimen demonstrates (A) abundant extracellular mucin with minimal cellularity, (B) extensive necrosis with numerous non-viable tumor cells, and (C) rare viable tumor cells. Areas highlighted within the red circle.

To establish a definitive diagnosis, a percutaneous liver biopsy was performed by interventional radiology. Histopathologic examination demonstrated abundant extracellular mucin with sparse viable tumor cells, consistent with mucinous adenocarcinoma (Figure 3). Based on imaging and metastatic involvement, the disease was staged as stage IV.

Treatment considerations

Given extensive hepatic and nodal metastases, the disease was deemed unresectable by surgical oncology. Systemic chemotherapy was recommended by medical oncology. For advanced biliary tract malignancies, combination therapy with gemcitabine and cisplatin is considered first-line treatment; however, lack of insurance coverage delayed initiation of therapy. Despite preserved functional status at diagnosis, the overall prognosis was poor given the metastatic stage IV mucinous adenocarcinoma.

Clinical deterioration and re-presentation

Two weeks following initial evaluation, the patient re-presented with worsening abdominal distension and melena. Upper endoscopy identified a partially obstructing mass infiltrating the duodenum, and an endoscopic stent was placed for palliation of obstruction.

During this admission, he developed acute kidney injury requiring initiation of hemodialysis. His course was further complicated by sepsis, spontaneous bacterial peritonitis, and severe anemia. Despite aggressive supportive management, he became profoundly hypotensive, requiring vasopressor support. He progressed to multi-organ failure and died 48 hours after readmission.

## Discussion

Mucinous adenocarcinoma of the gallbladder is a rare and aggressive subtype, accounting for approximately 2-3% of all gallbladder carcinomas and defined histologically by more than 50% extracellular mucin [7]. It is frequently diagnosed at advanced stages due to its asymptomatic progression [9], with poor prognosis attributed to its rapid dissemination and limited response to conventional therapies [8]. The aggressive clinical behavior of this subtype is thought to be related in part to the abundant extracellular mucin, which may facilitate tumor spread and contribute to delayed detection by obscuring malignant cells on cytologic evaluation.

This case exemplifies the typical presentation and challenges associated with this malignancy. At diagnosis, the patient had extensive liver and retroperitoneal lymph node involvement, consistent with known patterns of metastasis for mucinous adenocarcinoma of the gallbladder [10]. Rapid clinical deterioration followed, including duodenal obstruction, acute kidney injury, and eventual multi-organ failure, all occurring within two weeks of diagnosis, highlighting the fulminant nature of this disease, even in patients with initially preserved functional status [11]. This rapid decline likely reflects a combination of advanced tumor burden, aggressive tumor biology, and delayed initiation of systemic therapy.

Diagnostic complexity was heightened by the presence of abundant extracellular mucin, which can obscure tumor cells and delay histopathologic confirmation [7]. In this case, the diagnosis was only achieved through liver biopsy after ascitic fluid cytology revealed malignancy. Importantly, this highlights a key diagnostic pitfall: in patients presenting with malignant ascites and imaging findings suggestive of hepatobiliary malignancy, inconclusive or nonspecific cytology should not delay tissue biopsy. Accurate staging is crucial to inform surgical decision-making, particularly in aggressive subtypes such as this [12].

Given the presence of malignant ascites and hepatic lesions, the differential diagnosis included primary hepatobiliary malignancy, metastatic gastrointestinal carcinoma, and cholangiocarcinoma. While imaging suggested a gallbladder primary, definitive distinction required histopathologic confirmation. The low serum-ascites albumin gradient supported a non-portal hypertensive etiology, increasing suspicion for malignancy. In this case, low-SAAG ascites with normal liver function tests and elevated carcinoembryonic antigen (CEA) further narrowed the differential toward peritoneal malignancy rather than portal hypertension, prompting early tissue biopsy. In similar clinical scenarios, integrating imaging findings with ascitic fluid analysis and early tissue diagnosis is essential to avoid delays in management.

While similar cases have reported incidental diagnosis during evaluations for acute cholecystitis [11], or associations with precursor lesions such as mucinous cystic neoplasms and intracystic papillary neoplasms [13], no such precursor was identified here. This aligns with the tendency for mucinous adenocarcinomas to present without obvious antecedent pathology in advanced stages [14].

The patient's recent migration from Venezuela, a region endemic for *Salmonella Typhi *and *Helicobacter pylori* [4,5], raises important considerations. Chronic infection and inflammation are established risk factors for gallbladder carcinoma [2], and limited access to healthcare during prolonged migration may have delayed detection. This context underscores the relevance of adopting more tailored screening strategies for migrant populations, especially from regions with higher exposure to oncogenic pathogens [6]. Additionally, barriers such as a lack of insurance coverage may further delay timely diagnosis and initiation of therapy, as illustrated in this case.

Despite recommendations for systemic chemotherapy, the lack of insurance coverage delayed treatment. The uniformly poor outcome, even with planned intervention, mirrors findings from prior studies that report short survival times in advanced cases despite aggressive management [1,3]. The reported series describes median survival durations of approximately three to six months in mucinous variants, underscoring their aggressive clinical course and limited responsiveness to systemic therapy. This case further emphasizes how delays in treatment initiation, even when brief, may significantly impact outcomes in rapidly progressive malignancies.

This case reinforces the importance of heightened clinical suspicion, especially in patients presenting with nonspecific systemic symptoms and risk factors related to chronic inflammation or geographic origin. In clinical practice, early cross-sectional imaging and prompt biopsy should be prioritized in patients with low-SAAG ascites and systemic symptoms, even in the absence of definitive cytologic confirmation. Early detection remains the most promising avenue for improving outcomes in mucinous adenocarcinoma of the gallbladder - a malignancy that is both diagnostically elusive and rapidly fatal if missed.

This case highlights several key take-away lessons. Low-SAAG ascites should raise suspicion for an underlying malignant process until proven otherwise, particularly in patients without cirrhosis or evidence of portal hypertension. In addition, ascitic fluid cytology may be falsely non-diagnostic in mucinous malignancies because these tumors often yield scant cellular material. Therefore, when clinical suspicion for malignancy persists despite inconclusive cytology findings, early tissue diagnosis with image-guided biopsy is essential to avoid delays in diagnosis and guide appropriate management.

## Conclusions

Mucinous adenocarcinoma of the gallbladder is a rare and highly aggressive malignancy, often diagnosed at advanced stages due to its silent early course. Defined by abundant extracellular mucin, it is associated with rapid progression, frequent hepatic and peritoneal metastases, and limited responsiveness to standard therapies. This case highlights the diagnostic challenges and fulminant clinical course of this disease, including advanced presentation, rapid deterioration, and poor therapeutic outcomes despite supportive care. In patients presenting with unexplained ascites, particularly those from regions with endemic risk factors, clinicians should maintain a high index of suspicion for gallbladder malignancy. Early cross-sectional imaging and prompt tissue diagnosis are critical, even when initial cytology is inconclusive. Given the uniformly poor prognosis, timely recognition remains essential to improving outcomes in this rare and lethal disease.
